# Establishing a Multidisciplinary Context for Modeling 3D Facial Shape from DNA

**DOI:** 10.1371/journal.pgen.1004725

**Published:** 2014-11-06

**Authors:** Peter Claes, Mark D. Shriver

**Affiliations:** 1 Medical Image Computing, ESAT/PSI, Department of Electrical Engineering, Medical Imaging Research Center, KU Leuven & UZ Leuven, iMinds-KU Leuven Future Health Department, KU Leuven, Leuven, Belgium; 2 Department of Anthropology, Penn State University, University Park, Pennsylvania, United States of America; Seattle Children's Research Institute, United States of America

In their perspective piece on Claes et al. [1], Hallgrimsson and colleagues [2] make some points worthy of discussion, but do so largely in the context of a series of strong opinions that they incorrectly attribute to us. The pervasive straw man that is set up in the title and throughout their piece is that we think faces are simple traits, and that predicting facial shape from genotype is already practicable, consequentially overreaching the science. The point of our statement, quoted by these authors, “…*our methods provide the means of identifying the genes that affect facial shape and for modeling the effects of these genes to generate a predicted face*.” was to highlight the conceptual and methodological advances reported in that work (more on this below). The very next and final sentence of Claes et al., 2014 [1] frames the context of this sentence and is what we meant and by which we continue to stand, and reads, “*Although much more work is needed before we can know how many genes will be required to estimate the shape of a face in some useful way, and many more populations need to be studied before we can know how generalizable the results are, these results provide both the impetus and analytical framework for these studies.*” This concluding sentence clearly emphasizes that additional work is required and that we only claim to have provided a methodological framework and motivation. In a recent paper [3], we investigated a means of combining the effects of independent factors (namely, sex, genomics ancestry, and genotypes for the 24 single nucleotide polymorphisms (SNPs) from Claes et al.) into a single predicted face. We also explored considerations for how to judge the accuracy of these predicted faces. In short, although we find that sex and ancestry provide much more precision in estimating facial shape from these data, the 24 SNPs do add a small, but statistically significant, level of improvement in facial distinctiveness.

Although it remains to be seen how many alleles and loci affecting normal-range variation in facial features will be discovered, we are encouraged not only by the results presented in Claes et al. but by five rather common observations that are slowly, but surely being formally supported using modern morphometric methods: 1) identical twins are strikingly similar [4], 2) genetic relatives often show particular distinctive features [5], 3) conditions of atypical facial development are often distinctive and easily recognizable [6], 4) human population groups show observable differences [7], [8], and 5) men and women are facially distinctive [9], [10]. Despite the complexity of craniofacial development and the largely unknown mechanisms by which genetic variation affects facial features, these observations compellingly support the assertion that at least some genetic variants have consistent and thus predicable effects on the human face. Such a connection can provide sufficient impetus to apply human genetics methods to both discover which alleles and loci affect variation in the face and to attempt to model facial phenotype from genotype [11]–[13].

**Figure 1:**
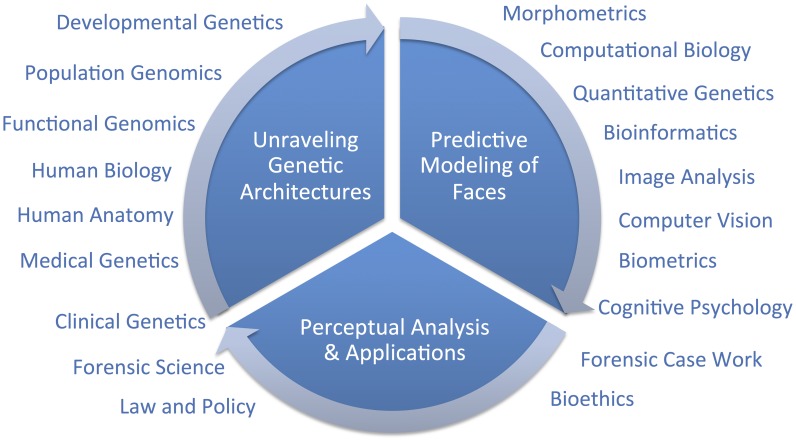
Diagram of a framework for research on modeling facial features from DNA.

Hallgrimsson twice cites one genome-wide association study (GWAS) on facial features [14] as evidence that the *SHH* gene plays no role in normal-range facial features and, because these authors found so few genes, as evidence that the genetic architecture of facial variation has a “very complex architecture.” Although these two points may well be proven true in time, negative evidence from one study is not very compelling support for either conclusion. Although we are cautious of strong conclusions based on analogies with other traits, such as the coronary heart disease example presented by Hallgrimsson, we do expect that different genetic and genomic [15] methods will be useful in identifying different types of variants. For example, rare variants with large effects, like those causing Mendelian conditions presenting with atypical craniofacial development, will most likely be discoverable using linkage analysis in families [16]. Alternatively, common alleles with smaller effect sizes will likely be easier to map using genetic association [17]. Alleles leading to facial differences between populations can be specifically targeted and thus most efficiently identified using admixture mapping [18]. There are a number of other sources of information beyond human–genetic methods that can and should contribute to facial feature gene identification efforts (Figure 1). Recent work by Hallgrimsson's group, for example, provided an interesting combination of functional genomic and animal model approaches using the mouse. Ideally, researchers will emerge who can make the most of several types of information to help understand the developmental genetic architecture of the human face. Indeed, the face is complex and we fully expect that a combination of all of these efforts will most constructively contribute to a more complete understanding of both its evolution and development.

One key aspect of facial research is how to systematically measure and model facial variation. In most prior facial feature mapping analyses, researchers focused on using individual interlandmark distances and principal component scores as traits [14], [19]–[21]. The primary drawback of these univariate approaches is that the response variables used represent only either some arbitrary or a priori subset of the total facial variation, which will not necessarily correspond to the facial effects of genes or any other factors. Additionally, univariate methods are statistically underpowered when used to map multivariate traits in GWAS [22], adding, for example, a layer of multiple testing reducing statistical power by a factor equal to the number of traits analyzed. Although most of the normal-range gene mapping studies have focused on univariate analyses, one recent paper used dense-correspondence based methods, which allow all parts of the face to be modeled together [23]. Dense correspondence methods have proven useful in investigating conditions of atypical facial development [24] and can be used to create average or consensus faces for cases and controls or, as demonstrated by Peng et al., by genotype. Although these genotype-average faces do allow any part of the face to be affected, it is currently unclear how to condition for confounding variables in these analyses, or how to accommodate continuously distributed independent factors, like genomic ancestry. Peng and colleagues overcame these limitations by focusing on a study population showing limited variation in genomic ancestry and by stratifying their analyses by sex, in effect matching males with males and females with females.

The approach we explored in Claes et al. is fundamentally different from the other methods being used to study human facial variation and facilitates both conditioning for confounding variables and the inclusion of all facial regions. Briefly, we applied partial least squares regression (PLSR) and multidimensional scoring in a novel forced imputation framework. This approach allows any set of facial regions to be combined into a single numerical score of that factor's effect on each face. These scores are essentially the predicted value of the independent (predictor) variable, e.g., sex, genomic ancestry, or genotype, given the relationship between facial variation and that variable observed in the sample. We called this new type of variable the response-based imputed predictor (RIP) variable and, given empirically observed improvements through multiple iterations, have called the method, generally, bootstrapped response-based imputation modeling (BRIM). The ability of BRIM to model facial sex and facial ancestry was assessed using a series of analytical experiments and human perception experiments [1].

Additionally, univariate methods provide no obvious means for visualizing facial modeling analyses as images. As shown in Claes et al., such images can be used in post hoc comparisons between normal-range effects and clinically significant effects [1]. Visualizing the effects also opens the door for systematic transformations of particular faces, which could be useful in experiments on the psychology of facial perception. Finally, without a means of visualizing the effects of genes and other factors, methods for assembling composite faces, like the one explored in our recent paper [3], would not be possible.

The important question that remains is, what is a suitable scientific context for modeling 3D facial shape from DNA? We do share Hallgrimsson and colleagues' perspective that when publishing novel scientific methods, it is important to establish reasonable expectations to policymakers and the public. The full context will only be known in time; overpromising results is certainly not the right framework for progress, but neither is diminishing novel synthetic efforts. Unraveling the genetic architecture of facial morphology is only one aspect of a comprehensive predictive modeling effort. The creation of usefully accurate DNA-based facial composites, as discussed in [3], involves at least two other aspects which are also quite multidisciplinary; namely, 1) predictive modeling of faces, and 2) perceptual analysis and applications. In the figure, we diagram these three primary components and indicate broadly which are some of the fields that can, and should, be drawn on to address these three components. We believe that the most constructive, and thus useful, context for facial feature genetics will be possible after adopting a multidisciplinary point of view.
